# New indicator *Escherichia coli* strain for rapid and accurate detection of *supF* mutations

**DOI:** 10.1186/s41021-020-00167-x

**Published:** 2020-09-22

**Authors:** Ruriko Fukushima, Tetsuya Suzuki, Hiroyuki Kamiya

**Affiliations:** Graduate School of Biomedical and Health Sciences, Hiroshima University, 1-2-3 Kasumi, Minami-ku, Hiroshima, 734-8553 Japan

**Keywords:** *supF* mutation assay, Indicator *Escherichia coli* strain, *gyrA*, *rpsL*

## Abstract

**Background:**

The *supF* gene of *Escherichia coli* is useful for forward mutation analysis in bacterial and mammalian cells used in mutagenesis and DNA repair studies. Indicator *E. coli* strains, such as KS40/pOF105, have been used to analyze *supF* mutations. However, KS40/pOF105 is not enough to select *supF* mutants on nutrient-rich agar plates. Therefore, in this study, a new indicator *E. coli* strain for rapid and accurate detection of *supF* mutations was developed.

**Results:**

The *gyrA* and *rpsL* genes with an amber mutation were integrated into the chromosomal DNA of *E. coli* KS40 to produce a new indicator strain, RF01. RF01 cells transformed by the wild-type *supF* gene were sensitive to nalidixic acid and streptomycin on LB agar plates. *supF* mutant frequencies and mutation spectra in RF01 were similar to those in KS40/pOF105. In addition, some mutations in *supF* were only detected in RF01.

**Conclusion:**

RF01 is a new and useful indicator *E. coli* strain for analyzing *supF* mutations.

## Background

The *supF* gene of *Escherichia coli* codes for an amber suppressor transfer RNA (tRNA) that translates the amber codon (UAG) into tyrosine [1, 2]. *supF* mutations stop translation of genes with an amber mutation. On the basis of this property, *supF*-bearing plasmids have been developed to study chemical mutagenesis and DNA repair mechanisms by using selectable marker genes with an amber mutation in host bacteria [3–6].

*E. coli* MBM7070 has an amber mutation in *lacZ*, and *supF* mutants can be identified by colorimetric screening using 5-bromo-4-chloro-3-indolyl-β-D-galactopyranoside (X-gal) [6]. However, it is difficult to select white mutant colonies from a large number of wild-type (WT) blue colonies and accurately determine low-level mutant frequencies in MBM7070. Akasaka et al. (1992) established *E. coli* KS40/pKY241 to overcome this difficulty [7]. KS40 is a nalidixic acid–resistant (*gyrA*) MBM7070 derivative, and pKY241 is a plasmid bearing the *gyrA* gene with an amber mutation. WT *supF* (*supF*^+^)-transformed KS40/pKY241 cells are sensitive to nalidixic acid, and *supF* mutants are selected as white colonies on nalidixic acid- and X-gal-containing agar plates. However, this antibiotic system does not work properly for selecting *supF* mutants, and false-positive mutant (blue) colonies are found on selection agar plates. To improve this antibiotic system, Obata et al. (1998) developed the indicator strain *E. coli* KS40/pOF105 for positive screening of *supF* mutants in order to measure low-level mutant frequencies [4], and KS40/pOF105 has been used to analyze mutations generated in bacterial and mammalian cells [8–20]. KS40 is also deficient in *rpsL* in addition to *gyrA*, and the pOF105 plasmid contains structural *gyrA*^*am*^ and *rpsL*^*am*^, which have an amber mutation in one of their tyrosine codons. Therefore, the *supF*^+^ plasmid transforms KS40/pOF105 to nalidixic acid- and streptomycin-sensitive and β-galactosidase-positive cells. In contrast, KS40/pOF105 cells remain resistant to nalidixic acid and streptomycin and β-galactosidase-negative when mutant *supF* (*supF*^−^) plasmids are introduced. Therefore, transformants with *supF*^−^plasmids form white or faint-blue colonies on nalidixic acid-, streptomycin-, and X-gal-containing selection agar plates. *supF* mutants can be successfully selected by dual antibiotic system on minimal agar plates [4]. However, some blue false mutant colonies, which are transformed by *supF*^+^ plasmids, often appear when selection agar plates contain a nutrient-rich medium and X-gal [15].

We hypothesized that *rpsL*^*am*^ expression from pOF105 is suppressed in nutrient-rich media containing tryptophan since the expression of *rpsL*^*am*^ is regulated under the tryptophan promoter. When minimal medium is used, as previously described [4], both nalidixic acid and streptomycin effectively select *supF* mutants. In contrast, in a nutrient-rich medium, only nalidixic acid selection might be effective, and spontaneous mutations in genes involved in nalidixic acid dynamics in *E. coli* or *gyrA*^*am*^ in pOF105 might allow *supF*^+^ colony formation. The culture time until colony formation on minimal agar plates is commonly longer compared to nutrient-rich agar plates. In addition, the preparation of minimal agar plates is more complex than that of LB agar plates consisted of nutrient-rich because many components must be added after autoclaving.

In this study, a new indicator *E. coli* strain allowing double selection by nalidixic acid and streptomycin on nutrient-rich agar plates and the rapid and accurate detection of *supF* mutations was established.

## Materials and methods

### Materials

Oligodeoxynucleotides were purchased from Integrated DNA Technologies (Coralville, IA, USA), Hokkaido System Sciences (Sapporo, Japan), and Sigma Genosys Japan (Ishikari, Japan) (Table 1). U2OS cells were obtained from ATCC (ATCC HTB-96). KS40 and KS40/pOF105 were kindly provided by Professor Tatsuo Nunoshiba of International Christian University [4].

**Table 1:** Oligodeoxynucleotide sequences used in this study

Name	Sequence (5′ to 3′)
repA(A56V) S	ATAACCAATACGTTCAGATGATGAA
repA(A56V) AS	TTCATCATCTGAACGTATTGGTTAT
pBAD-CYC(X) Fw	TCAGATAAAATATTTTGCATAATGTGCCTGTCAAA
pBAD-(MCS) Rv	TGCAGAAGCTTCCTCCTGTTAGC CCAAAAAAACGGGTATGGAG
rrnB term-(pBAD) Fw	GAGGAAGCTTCTGCAGCTCGAG TGCCTGGCGGCAGTAGCGCG
rrnB term-CYC(D) Rv	GCAAATTCGACCCGG AAGGCCCAGTCTTTCGACTG
Red (pBADMCS-H) Fw	GGGCTAACAGGAGGA TTATAAAAAATGGATATTAA
Red (pBADMCS-H) Rv	CACTCGAGCTGCAGA ATTCTTCGTCTGTTTCTACT
rpsL pro SalI Fw1	CCAGTCGACTGGCCTGGTGATGGCGGGAT
rpsL Lower3	CACGAGTACATACGCCACGT
gyrA SgrAI Fw	TATTCTGCTGACGCACGGCATTCATTGGCACTTCT
gyrA SgrAI Rv	AGAAAAAAGGCTGCACCTTGTGTATAGCCAGCCAT
pCpGfree MAR(−) VF	TGTGGTATGGAATTC TTAAAATCAGCAGTTCAACCTGTT
pCpGfree MAR(−) VR	CTCCTGCAGGAATTC TTAAAACAGTAGTTGACAATTAAACAT
arsB(500) Fw	GGCAGCCGAAAGGTTTAGGC
arsB(500) Rv	CCATCAATGGACAACGCGCC
HL-CYC Fw	AAACAAAAAAAACCCCGCTTCGGCGGGGTTTTTTTTGCACCTGAAGTCAGCCCCAT
HL-CYC Rv	AAACAGCGAAAAAACCCCGCCCTGTCAGGGGCGGGGTTTTTTGCGCGAGCGTAGCGAGTCAGTGA
supF RM Fw	CGCCGTCTCGGTTATTGTCTCATGAGCGG
supF RM Rv	GCCCGTCTCAGCTCTTGATCCGGCAAACA
supFcomp RM Fw	GCCCGTCTCAGAGCTACCAACTCTTTTTC
supFcomp RM Rv	CGCCGTCTCATAACCCTGATAAATGCTTC

### Construction of a temperature-sensitive λ-red operon–expressing plasmid

To produce temperature-sensitive pMW119ts, the alanine 56 substitution to valine (167 C ➔ T) in *repA* was introduced into pMW119 (Nippon Gene, Tokyo, Japan) using the QuikChange Site-Directed Mutagenesis Kit (Agilent Technologies, Santa Clara, CA, USA) and primers repA(A56V) S and repA(A56V) AS. The *araBAD* promoter and the *rrnB* terminator were amplified by PCR using *E. coli* BL21(DE3) genomic DNA as a template and two primer sets: pBAD-CYC(X) Fw and pBAD-(MCS) Rv and rrnB term-(pBAD) Fw and rrnB term-CYC(D) Rv. Next, to construct pBAD-MCS, the PCR fragments were combined with a short fragment of pACYC184 (Nippon Gene) digested with *Xba* I and *Drd* I using the GeneArt Seamless Cloning and Assembly Enzyme Mix (Thermo Fisher Scientific, Waltham, MA, USA). The λ-red operon was amplified by PCR using λDNA (New England Biolabs, Ipswich, MA, USA) as a template and the primer set Red (pBADMCS-H) Fw and Red (pBADMCS-H) Rv, and the PCR fragments were then joined with *Hin*d III-digested pBAD-MCS. Next, the λ-red operon expression cassette from the *araBAD* promoter to the *rrnB* terminator was amplified and ligated into the *Sma* I site of pMW119ts in the reverse direction of *lacZα*. Finally, the *E. coli* KS40 strain transformed by the resultant plasmid was cultured at 30 °C in a medium containing 0.2% arabinose, and competent cells were prepared for electroporation.

### Establishment of the *E. coli* RF01 strain

Structural *gyrA*^*am*^ and *rpsL*^*am*^ were integrated into *E. coli* chromosomal DNA using the λ-red recombination system, as previously described with slight modifications [21, 22].

**Fig. 1:**
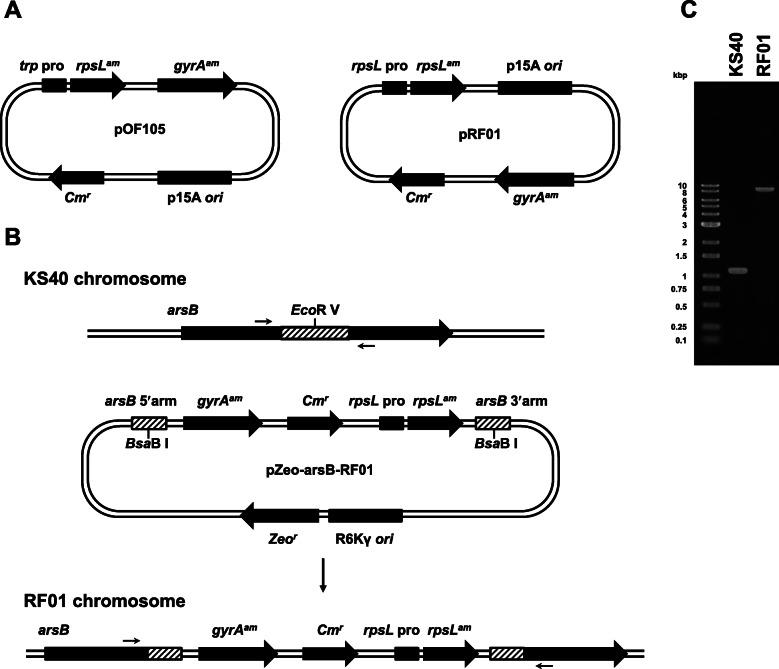
**a** pOF105 and pRF01 plasmid maps. **b** Knock-in strategy of *gyrA*^*am*^ and *rpsL*^*am*^ into the *arsB* gene locus by λ-red recombination and (**c**) genotyping of the *arsB* gene locus in KS40 and RF01. The small arrows above/below the genes in (**b**) indicate the primers used for genotyping by PCR, which is shown in (**c**). The chain lengths of amplicons derived from KS40 and RF01 genomic DNAs were 1115 and 8391 bp, respectively

The *rpsL* promoter was amplified using pSSW [23] as a template and the primer set rpsL pro SalI Fw1 and rpsL Lower3. The amplified fragment was digested with restriction enzymes *Sph* I and *Sal* I and ligated with the large pOF105 fragment also digested with *Sph* I and *Sal* I, and the plasmid was digested with *Bam*H I and *Bgl* II, followed by self-ligation to remove *gyrA*^*am*^. Subsequently, the gene amplified using pOF105 as a template and the primer set gyrA SgrAI Fw and gyrA SgrAI Rv was reintroduced into the *Sgr*A I site using the GeneArt Seamless Cloning and Assembly Enzyme Mix to produce pRF01 (Fig. 1a).

The DNA region containing the Zeocin-resistance gene plus R6Kγ *ori* was amplified by PCR using pCpGfree-mbSeap [24] as a template and the primer set pCpGfree MAR(−) VF and pCpGfree MAR(−) VR. It was then joined with the PCR-amplified *arsB* fragment using BL21(DE3) genomic DNA as a template and the primer set arsB (500) Fw and arsB (500) Rv in the same direction as the Zeocin-resistance gene. To construct pZeo-arsB-RF01 (Fig. 1b), the resultant plasmid was digested with *Eco*R V and ligated with PCR fragments amplified using pRF01 as a template and the primer set HL-CYC Fw and HL-CYC Rv. Next, 100 ng of *Bsa*B I-digested pZeo-arsB-RF01 was electroporated into λ-red-expressing KS40 competent cells and selected on LB agar plates containing 5 μg/mL chloramphenicol at 37 °C. Finally, the genotype and ampicillin sensitivity of chloramphenicol-resistant *E. coli* was confirmed, and the *E. coli* strain was named RF01 (Fig. 1b and c).

### Introduction of random mutations into *supF*

*supF* was amplified by random mutagenesis PCR using pZ189-T_E107K/D402E [17] as a template and the primer set supF RM Fw and supF RM Rv, as previously described [25]. Briefly, a 50 μL-reaction mixture containing 1 × Taq buffer (Toyobo, Osaka, Japan), 0.2 mM dATP, 0.2 mM dGTP, 1 mM dCTP, 1 mM dTTP, 0.5 mM MnCl_2_, 0.3 μM each primer, 50 ng of pZ189-T_E107K/D402E, and 2.5 units of *Taq* DNA polymerase (Toyobo) was amplified by PCR for 35 cycles of 94 °C for 30 s, 55 °C for 30 s, and 72 °C for 45 s. The pZ189-T_E107K/D402E backbone was amplified using the primer set supFcomp RM Fw and supFcomp RM Rv under the standard PCR condition using the KOD One PCR Master Mix (Toyobo). Both DNA fragments were digested with *Esp*3 I and combined, and the ligation product was introduced into *E. coli* DH10B. Finally, ~ 10^5^ colonies on ampicillin-containing agar plates were harvested, followed by plasmid extraction, and the plasmid was named pZ189-RM.

### Comparison of recovery from electroporation of *E. coli* RF01 vs. KS40/pOF105

A mixture of equal amounts of pZ189-T_E107K/D402E and pZ189-T_E107K/D402E (122 T) [17] was electroporated into RF01 and KS40/pOF105 cells. Immediately after the addition of SOC medium and after a 1-h recovery, the cultures were seeded on LB agar plates containing 150 μg/mL ampicillin, 30 μg/mL chloramphenicol, and 80 μg/mL X-gal.

### *supF* mutation analyses

pZ189-T_E107K/D402E was transfected into human U2OS cells using Lipofectamine 2000 according to the manufacturer’s instructions, and the plasmids were extracted as previously described [26]. The extracted plasmids or mixtures of pZ189-T_E107K/D402E and pZ189-RM were introduced into RF01 and KS40/pOF105 cells, and *supF* mutant frequencies were calculated. Finally, mutation spectra of the plasmid from *E. coli* strains transformed by pZ189-RM were analyzed.

The colony sizes are visually judged based on the sizes of colonies on titer plates.

### Statistical analysis

Statistically significant differences in blue-to-white colony ratios and *supF* mutant frequencies between RF01 and KS40/pOF105 were examined by Student’s *t*-test. The level of statistical significance was set at *P* < 0.05.

## Results

### Establishment of a new indicator strain for *supF* mutation analyses

**Fig. 2:**
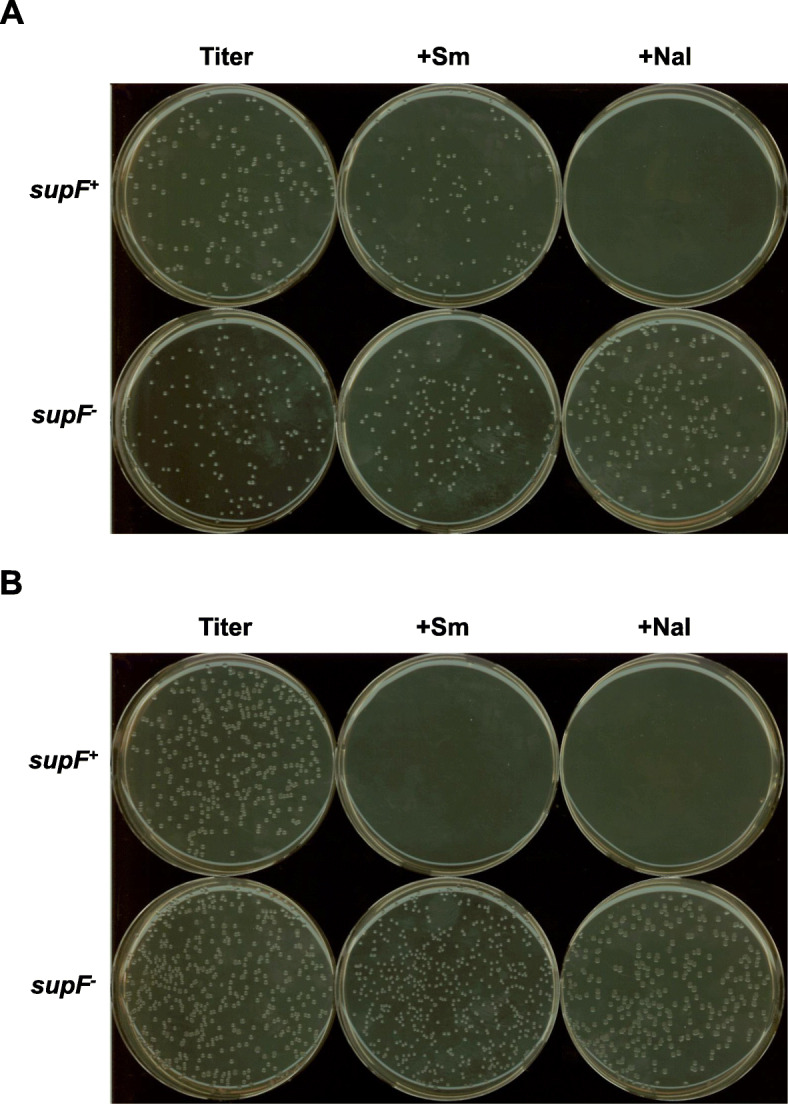
Nalidixic acid and streptomycin sensitivity of (**a**) KS40/pOF105 and (**b**) RF01 cells transformed with the plasmids *supF*^+^ (pZ189-T_E107K/D402E) and *supF*^−^ (pZ189-T_E107K/D402E (122 T)). Sm, streptomycin; Nal, nalidixic acid

We transformed KS40/pOF105 cells with a *supF*^*+*^-bearing plasmid and spread them onto LB agar plates containing streptomycin to test our hypothesis that antibiotic sensitivity is ineffective when nutrient-rich agar plates are used. The numbers of colonies on agar plates with and without streptomycin were similar, although the colony size was smaller on selection agar plates than on titer plates (Fig. 2a). This result indicated that selection with the antibiotic is ineffective when KS40/pOF105 and streptomycin-LB agar plates are used.

To enable selection by streptomycin in nutrient-rich media, such as LB medium, the tryptophan promoter in pOF105 was replaced with the *rpsL* promoter for constitutive *rpsL*^*am*^ expression in tryptophan-containing media. The constructed plasmid, pRF01 (Fig. 1a), was introduced into KS40. KS40/pRF01 cells transformed with a *supF*^*+*^-bearing plasmid showed sensitivity to both nalidixic acid and streptomycin (data not shown). However, the colony size of *supF*^*+*^ plasmid-bearing KS40/pRF01 cells was smaller than that of *supF*^*+*^ plasmid-bearing KS40/pOF105 cells and *supF*^*−*^ plasmid-bearing KS40/pRF01 cells on LB titer plates (data not shown). This result indicated that high-level *rpsL* expression from multiple plasmid copies negatively affects growth.

**Fig. 3:**
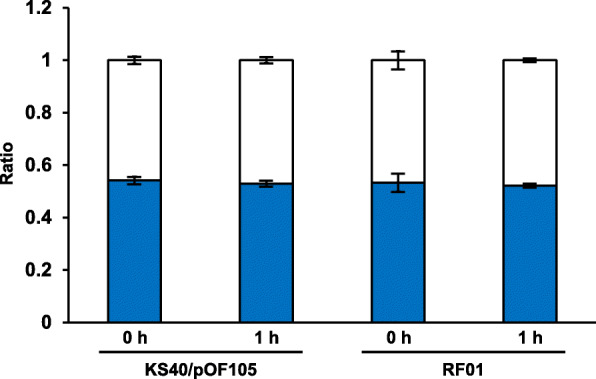
Comparison of blue-to-white colony ratios for KS40/pOF105 and RF01 cells. Equal amounts of *supF*^+^ (pZ189-T_E107K/D402E) and *supF*^−^ (pZ189-T_E107K/D402E (122 T)) plasmids were electroporated into KS40/pOF105 and RF01, and treated bacteria were placed on LB agar plates containing X-gal 0 and 1 h postincubation in SOC medium. Blue and white bars indicate blue and white colony ratios. Data are represented as the means ± SD (standard deviation) of three independent experiments. There were no significant differences between 0 and 1 h postelectroporation and between RF01 and KS40/pOF105

A new *supF* mutation indicator strain, RF01, was then established by integrating *gyrA*^*am*^ and *rpsL*^*am*^ into the *arsB* gene locus in the KS40 chromosomal DNA to reduce the copy number (Fig. 1b). The genotypes were confirmed by PCR (Fig. 1c, expected lengths of PCR products; KS40, 1115 bp; RF01, 8391 bp). RF01 cells transformed with a *supF*^*+*^-bearing plasmid showed sensitivity to nalidixic acid and streptomycin (Fig. 2b). In addition, the colony size of *supF*^+^-transformed RF01 cells on titer plates was not different from those of *supF*^+^-transformed KS40/pOF105 and *supF* mutant (122 G ➔ T)-transformed RF01 cells. The blue-to-white colony ratios were nearly identical for RF01 and KS40/pOF105 at 0 and 1 h postelectroporation for an ~ 1:1 mixture of WT and mutant plasmids (Fig. 3), indicating that the postelectroporation recovery rates were similar in RF01 and KS40/pOF105 regardless of the *supF* genotype and that neither strain had a potential bias in transformant growth during recovery culture.

### *supF* mutation detection capability of RF01 and KS40/pOF105

**Fig. 4:**
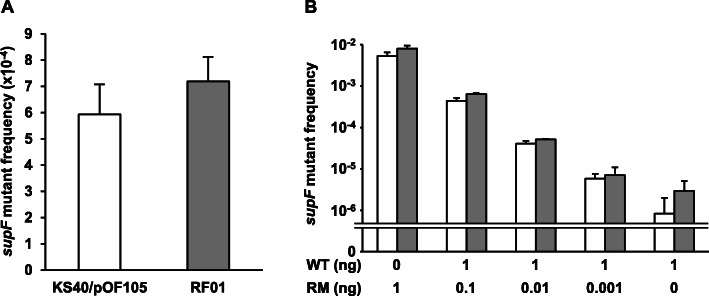
(**a**) *supF* mutant frequencies of plasmid replicated in U2OS cells and (**b**) mixtures of randomly mutagenized *supF* (pZ189-RM) and *supF*^+^ (pZ189-T_E107K/D402E) plasmids in KS40/pOF105 and RF01. pZ189-RM was mixed with pZ189-T_E107K/D402E at the ratios shown in (**b**). White and gray bars indicate KS40/pOF105 and RF01, respectively. Data are represented as the means + SD (standard deviation) of three independent experiments. There were no significant differences between RF01 and KS40/pOF105 in all the *supF* mutant frequencies

We then compared the *supF* mutation detection capability of RF01 and KS40/pOF105. The pZ189-T_E107K/D402E plasmid was transfected into human U2OS cells, and the replicated plasmid was recovered from the cells. The *supF* mutant frequencies (background mutant frequencies) were similar in both RF01 and KS40/pOF105 when the extracted plasmid was introduced into the two strains (Fig. 4a). In addition, we observed no blue (false mutant) colonies of RF01 expressing WT *supF* on selection agar plates.

**Fig. 5:**
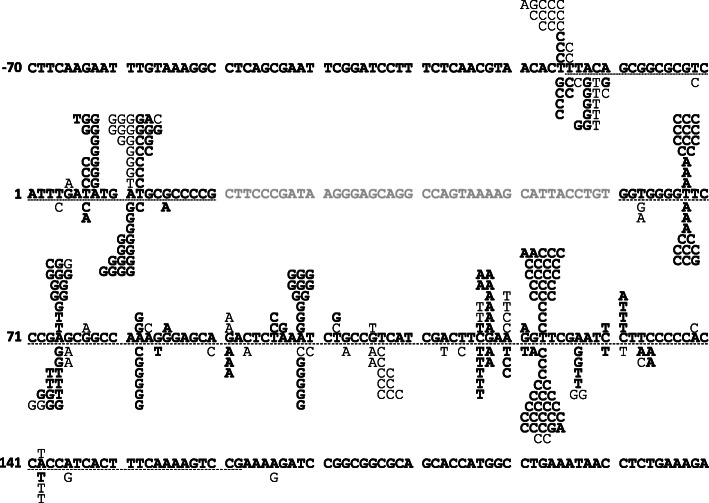
*supF* mutation spectra of pZ189-RM introduced into KS40/pOF105 and RF01. The upper strand sequence of *supF* is shown, and single base substitutions observed in *supF* mutants of KS40/pOF105 and RF01 are shown above and below the sequence, respectively. Dotted and dashed underlines represent promoter and *supF* tRNA coding sequences, respectively; gray font indicates the pre-tRNA sequence; thin letters indicate mutants that formed smaller colonies; and bold letters indicate mutants that formed normal-sized colonies

**Fig. 6:**
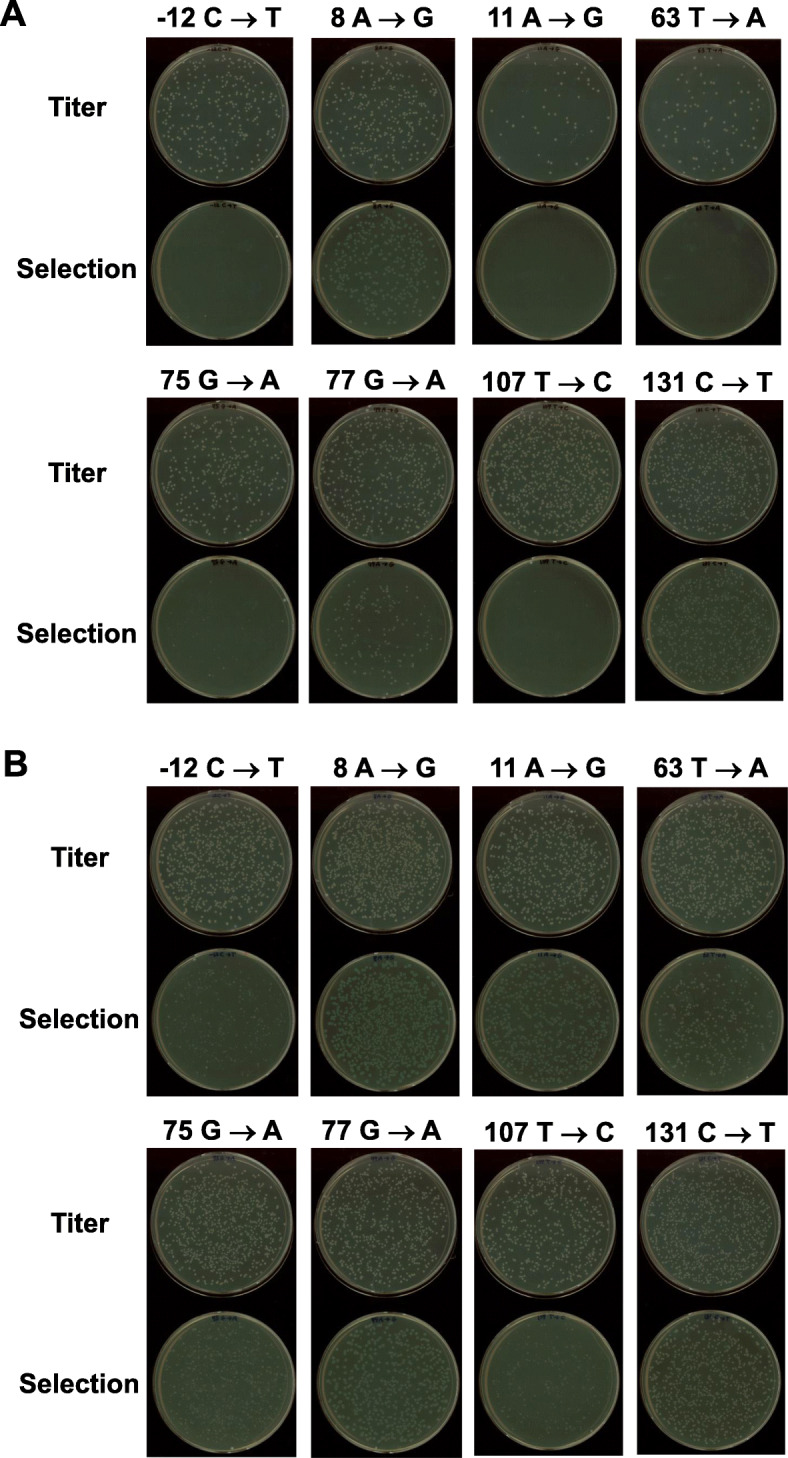
Colony formation potential of (**a**) KS40/pOF105 and (**b**) RF01 transformants with mutant *supF* plasmids on selection agar plates

In addition, we introduced pZ189-RM bearing randomly mutated *supF*. Mn^2+^ and imbalanced nucleotide conditions were used to induce random mutations in *supF*. pZ189-RM and WT pZ189-T_E107K/D402E were mixed in different proportions, and mutant frequencies and mutation spectra in RF01 and KS40/pOF105 were compared. The mutant frequencies of pZ189-RM plus pZ189-T_E107K/D402E were similar in RF01 and KS40/pOF105, depending on the pZ189-RM–pZ189-T_E107K/D402E ratio (Fig. 4b). We also analyzed *supF* mutation spectra in pZ189-RM-transformed mutants (Fig. 5). The mutation spectra were similar for RF01 and KS40/pOF105 cells. However, some mutations were observed in either RF01 or KS40/pOF105, and the colony sizes of some *supF* mutants seemed different between the two strains. Eight plasmids bearing this type of mutation (− 12 C ➔ T, 8 A ➔ G, 11 A ➔ G, 63 T ➔ A, 75 G ➔ A, 77 G ➔ A, 107 T ➔ C, and 131 C ➔ T) were introduced into RF01 and KS40/pOF105 cells. Plasmids containing mutations detected only in RF01 cells were introduced into KS40/pOF105 cells, and no or tiny colonies appeared on selection agar plates (− 12 C ➔ T, 63 T ➔ A, 75 G ➔ A, and 107 T ➔ C; Fig. 6a). In contrast, all *supF* mutants in RF01 formed colonies on selection agar plates (Fig. 6b), indicating that RF01 detects more types of *supF* mutations than KS40/pOF105.

## Discussion

This study established a new *supF* mutation indicator *E. coli* strain for rapid and accurate detection on nutrient-rich agar plates. *supF* mutant frequencies and mutation spectra were slightly different between KS40/pOF105 and RF01 (Figs. 4 and 5). In addition, the growth rates of RF01 postelectroporation of *supF*^*+*^ and *supF*^−^ plasmids were not significantly different (Fig. 3). These findings show that RF01 can be used for *supF* mutation analyses, similar to KS40/pOF105. Importantly, RF01, unlike KS40/pOF105, does not require minimal agar plates for mutant selection. The culture time to form colonies on minimal agar plates was 2 d, whereas that on LB agar plates was overnight (16 ~ 18 h) (data not shown). Nalidixic acid and streptomycin in LB agar plates completely inhibited the growth of RF01 cells carrying *supF*^*+*^, while a significant number of KS40/pOF105 cells containing WT *supF* appeared as blue colonies on LB agar plates containing nalidixic acid, streptomycin, and X-gal. KS40/pOF105 requires X-gal, an expensive reagent, for distinguishing between true and false mutant colonies, but RF01 does not. These findings show that the *supF* mutation assay is more rapid, accurate, and cost effective with RF01 cells. In addition, there are a few *supF* mutations detected only in RF01, and *supF* mutation frequencies in RF01 are slightly higher than those in KS40/pOF105. The *gyrA*^*am*^ copy number in RF01 is 1, so *gyrA*^*am*^ expression in RF01 should be lower than that in KS40/pOF105. Therefore, partially inactive *supF* mutations that are difficult to detect in KS40/pOF105 might be detected in RF01. Most of the mutations from the small colonies were found in the promoter of the *supF* gene, and the regions corresponding to the loops (except for the anticodon trinucleotide) and the central portions of stems of the cloverleaf structure of the *supF* tRNA. The mutations in the promoter possibly allow slight transcription and those in the loops and the central portions of stems would not completely disrupt the *supF* tRNA structure. Therefore, these mutations may lead to partial loss of *supF* function and consequently weak expression of the *gyrA* and *rpsL* genes with an amber mutation resulting in forming small colonies on selection agar plates.

## Conclusion

We developed a new indicator *E. coli* strain, RF01, for the rapid and accurate detection of *supF* mutations. RF01 is a novel and useful indicator for *supF* mutations.

## Data Availability

The datasets generated and/or analyzed during the current study are available from the corresponding authors on reasonable request.
